# Social Inequalities and Depressive Symptoms in Adults: The Role of Objective and Subjective Socioeconomic Status

**DOI:** 10.1371/journal.pone.0169764

**Published:** 2017-01-20

**Authors:** Jens Hoebel, Ulrike E. Maske, Hajo Zeeb, Thomas Lampert

**Affiliations:** 1 Unit of Social Determinants of Health, Department of Epidemiology and Health Monitoring, Robert Koch Institute, Berlin, Germany; 2 Unit of Mental Health, Department of Epidemiology and Health Monitoring, Robert Koch Institute, Berlin, Germany; 3 Department of Prevention and Evaluation, Leibniz Institute for Prevention Research and Epidemiology - BIPS, Bremen, Germany; University of Illinois at Urbana-Champaign, UNITED STATES

## Abstract

**Background:**

There is substantial evidence that lower objective socioeconomic status (SES)—as measured by education, occupation, and income—is associated with a higher risk of depression. Less is known, however, about associations between perceptions of social status and the prevalence of depression. This study investigated associations of both objective SES and subjective social status (SSS) with depressive symptoms among adults in Germany.

**Methods:**

Data were obtained from the 2013 special wave of the German Health Update study, a national health survey of the adult population in Germany. Objective SES was determined using a composite index based on education, occupation, and income. The three single dimensions of the index were also used individually. SSS was measured using the MacArthur Scale, which asks respondents to place themselves on a 10-rung ‘social ladder’. Regression models were employed to examine associations of objective SES and SSS with current depressive symptoms, as assessed with the eight-item Patient Health Questionnaire depression scale (PHQ-8 sum score ≥10).

**Results:**

After mutual adjustment, lower objective SES and lower SSS were independently associated with current depressive symptoms. The associations were found in both sexes and persisted after further adjustment for sociodemographic factors, long-term chronic conditions, and functional limitations. Mediation analyses revealed a significant indirect relationship between objective SES and depressive symptoms through SSS. When the three individual dimensions of objective SES were mutually adjusted, occupation and income were independently associated with depressive symptoms. After additional adjustment for SSS, these associations attenuated but remained significant.

**Conclusions:**

The findings suggest that perceptions of low social status in adults may be involved in the pathogenesis of depression and play a mediating role in the relationship between objective SES and depressive symptoms. Prospective studies are needed to establish the direction of effects and to address questions of causality.

## Introduction

Epidemiological research consistently shows a graded association between socioeconomic status (SES) and health: the lower people’s SES, the poorer their health and the greater their risk of chronic disease and premature death [1–4]. This socioeconomic gradient does not apply only to physical conditions and mortality, but also to mental health issues. In particular, anxiety and mood disorders, such as depression, are more prevalent in lower than in higher socioeconomic groups [5–7].

In studies on social inequalities in health, SES is traditionally determined based on information about educational qualifications, occupational positions, and income levels of individuals or households [8–10]. Researchers use these objective measures to classify people as belonging to a specific socioeconomic group. The use of multiple indicators in the measurement of SES originates back to Max Weber’s theory of social stratification, which emphasizes the multidimensional nature of social inequality [8, 10]. In social epidemiology, education, occupation, and income are regarded as the core dimensions of SES; each has a specific nature and latent content with specific implications for health [11–13]. However, a simultaneous examination of all the core dimensions in studies of socioeconomic inequalities in health is the exception rather than the standard. Geyer et al. [13] have pointed out that education, occupation, and income cannot be used interchangeably, as they reflect different phenomena and point to different mechanisms underlying social inequalities in health. With regard to mental health, educational attainment reflects and is related to non-material resources, such as cognitive abilities, knowledge, self-efficacy, attitudes, and values shaping mental-health-related behaviors, coping strategies, and the use of mental health services [13–15]. Occupation can affect mental health through exposure to psychosocial stressors related to lack of control at work, job strain, imbalance between work-related effort and reward, and low occupational social prestige [16–18]. The mental health implications of low income can also be stress-related and be caused by financial strain, resulting concerns about the future, and negative cognitions associated with low income-related social rank [19–21].

In addition to objective SES measures, the concept of subjective social status (SSS) was introduced to health research in 2000 [22] and has been increasingly used in epidemiological and health-related studies for nearly a decade [23, 24]. SSS assesses how individuals perceive their standing in the social hierarchy and with which status group they feel affiliated. It is thus an indicator of how people self-evaluate their access to socioeconomic resources in relation to other members of society and of related feelings of inequality between self and others [25, 26]. Research suggests that SSS is a product of social comparison processes and reflects individual perceptions of having fair or unfair opportunities in life [27, 28]. Therefore, SSS cannot simply be regarded as a proxy measure of SES, but rather as an indicator of subjective aspects of social stratification that complements traditional measures of SES. Although the concept of SSS is relatively new in health research, the idea that a person’s belief about his or her position in a status hierarchy does not necessarily correspond with the status accorded to him/her by others has a longer tradition in the social sciences [29, 30]. Even the early work of Richard Centers [31] demonstrated that people who are classified as belonging to a low socioeconomic group do not have to think of themselves as belonging to it.

The use of the SSS concept in health research is concomitant with the idea that perceptions of social status may have implications for health beyond the effects of objective socioeconomic factors. Hegar and Mielck [23] conducted a systematic review of studies investigating associations between SSS and health. They found 53 relevant studies published up to the year 2009; the earliest were published in 2000. Most of the studies were from the USA and had been published between 2007 and 2009; none of the studies were from Germany. This emphasizes how new the concept of SSS still is in health research—especially in Europe. Many of the studies included in the review revealed that low SSS is associated with several dimensions of ill-health, even after controlling for objective SES measures. A subsequent narrative review that included more recent data confirmed this conclusion [24]. For example, the World Mental Health Surveys showed inverse associations between SSS and several mental disorders after adjusting for objective socioeconomic factors [32].

This study examines socioeconomic inequalities in depressive symptoms in the adult general population of Germany. Compared with most other European countries, Germany has been in a better economic situation for the last few years. The German economy developed more positively and showed a faster rebound after the global financial crisis of 2007–09 [33]. The current unemployment rate is at its lowest since German reunification [34]. Correspondingly, people in Germany rate their social standing higher than people in other European countries [35]. As objective socioeconomic factors substantially determine people’s perceptions of their social standing, which in turn can independently predict changes in mental health [36], we investigated whether SSS is a potential mediator of the association between objective SES and depressive symptoms. As the social determinants of depression and the underlying mechanisms can differ by sex [37–39], this question was examined separately for men and women. In particular, we aimed to examine

1. whether the SSS of men and women in Germany is associated with depressive symptoms independently of their objective SES;
2. whether SSS plays a mediating role in the relationship between objective SES and depressive symptoms; and
3. the relative importance of the individual SES core dimensions (education, occupation, income) for depressive symptoms in men and women.

## Materials and Methods

### Study design and data collection

Our analyses were based on data obtained from the cross-sectional German Health Update (GEDA) study, a national health survey of the adult population in Germany. GEDA is part of the German-wide health monitoring system administered by the Robert Koch Institute (RKI) in Berlin [40]. The RKI is a federal institution within the portfolio of the German Federal Ministry of Health. The aim of the regularly conducted GEDA surveys is to provide current data on population health, health determinants, and use of health services [41].

For the present study, we used data from the 2013 special wave (GEDA 2013s), which was based on a two-stage stratified cluster sample. The target population was persons aged 18 years and older with permanent residence in Germany. In the first sampling stage, 100 communities were randomly selected from a list of all communities in Germany, stratified by federal state and urbanization grade. Sampling probabilities were proportional to the population size of the communities using the Cox procedure for controlled rounding [42]. In the second sampling stage, people with principal residence in the sampled communities were randomly drawn from the local population registers. The day of sampling was 20 November 2013.

Data were collected either by self-administered postal questionnaire or by self-administered web survey. The standardized questionnaires included questions about health status, health determinants, use of health services, and sociodemographic characteristics. A total of 4952 individuals aged 18 to 95 years had completed the survey by the end of June 2014. The proportion of web participants was 45%. According to the internationally used standard definitions of outcome rates for surveys [43], the ‘Response Rate 1’ was 20%. This so-called ‘minimum response rate’ represents the number of participants divided by the number of participants plus the number of non-participants plus all cases of unknown eligibility.

### Ethics and consent to participate

Data were collected exclusively by self-administered questionnaires. No physical examination or laboratory testing was performed; biological samples were not collected. The study was approved by The Federal Commissioner for Data Protection and Freedom of Information in Germany. Informed consent was obtained from all participants. Participants were informed about the goals and contents of the study, about privacy and data protection, and that their participation in the study was voluntary.

### Objective socioeconomic status

Objective SES was determined using a composite index developed for all surveys conducted within the national health monitoring system in Germany [44, 45]. The index includes information on education, occupation, and income. Education was assessed using the CASMIN educational classification, which takes into account information on respondents’ school-leaving and vocational qualifications [46]. Occupational status was determined using the International Socio-Economic Index of Occupational Status (ISEI) developed by Ganzeboom et al. [47]. It was operationalized as a household characteristic, i.e. the status value of the participant and the principal earner in the participant’s household were compared and the higher value was used. Where no person in the household was currently employed, the occupation pursued most recently was used. The occupational status variable was accordingly rather a measure of the occupational social prestige of the participants' household than a measure of the participants' individual occupational position. Income level was assessed via the net equivalent income; for this, household net income was adjusted for household size and age-specific needs of household members using the modified Organisation for Economic Co-operation and Development (OECD) equivalence scale [48]. This procedure made it possible to take account of the household composition to determine the participant's individual financial room for maneuver. To calculate the SES index, the three individual dimensions were transferred to metric subscales with a value range of 1.0 to 7.0. The point scores of the three subscales were then summed to compute a total index score ranging from 3.0 to 21.0. Table 1 presents the means, ranges, and standard deviations of the index and its subscales in GEDA 2013s. More details on the index and methods used in its construction can be found elsewhere [44, 45].

**Table 1:** Characteristics of the study population (n = 4,952; n_men_ = 2,183; n_women_ = 2,769).

	Total	Men	Women
	Mean (SD)	Mean (SD)	Mean (SD)
Age (18–95 years)	49.9 (18.1)	48.9 (17.7)	50.9 (18.5)
SES (score 3–21)	11.6 (3.9)	11.8 (4.1)	11.4 (3.7)
Education (score 1–7)	3.5 (1.7)	3.7 (1.8)	3.3 (1.6)
Occupation (score 1–7)	4.1 (1.5)	4.0 (1.6)	4.2 (1.3)
Income (score 1–7)	4.0 (1.9)	4.1 (1.9)	3.9 (1.9)
SSS (rung 1–10)	5.2 (1.7)	5.3 (1.8)	5.2 (1.6)

SD, standard deviation; SES, composite index of objective socioeconomic status; SSS, subjective social status.

The advantage of using a composite index of SES is that it enables the detection of cumulative effects of individual SES dimensions on a health outcome. However, the disadvantages are that the index masks the effects of the individual dimensions and conceals the relative importance of education, occupation, and income for the health outcome [49]. Therefore, we additionally used the subscales of the SES index to examine the associations of the individual SES dimensions with depressive symptoms. To estimate prevalence rates for depressive symptoms stratified by objective SES indicators, the SES index and its subscales were categorized into ‘low’ (quintile 1), ‘middle’ (quintiles 2–4), and ‘high’ (quintile 5) by quintiles of the whole population.

### Subjective social status

SSS was measured using a German version of the MacArthur Scale [26], a visual analogue scale that shows a picture of a 10-rung ‘social ladder’ and asks participants to indicate the rung on which they feel they stand. Originally, the instrument was developed by Adler et al. [22] to measure the SSS of people living in the United States. Since then, it has been translated into other languages and adapted to different countries [26, 50, 51] and has become an internationally established standard tool to measure SSS in health-related studies [23]. The German version showed adequate construct validity in a previous study [26]. To estimate prevalence rates for depressive symptoms according to SSS, we classified the SSS score as ‘low’ (quintile 1), ‘middle’ (quintiles 2–4), and ‘high’ (quintile 5).

### Depressive symptoms

The eight-item Patient Health Questionnaire (PHQ-8) depression scale was used to screen for current depressive symptoms. The scale showed good psychometric properties and screening accuracy in previous studies [52, 53] and is suitable for clinical use as well as for surveys of the general population [54]. The PHQ-8 assesses the presence and frequency of eight depressive symptoms (little interest or pleasure, depressed mood, sleep disturbances, tiredness or little energy, poor appetite or overeating, feelings of worthlessness or guilt, trouble concentrating, and psychomotor retardation or agitation) within the last 2 weeks, with the following possible answers: ‘not at all’ (score = 0), ‘several days’ (score = 1), ‘more than half the days’ (score = 2), or ‘nearly every day’ (score = 3). The scale showed adequate internal consistency in GEDA 2013s with a Cronbach’s alpha = 0.86, similar to that reported in the literature [52]. We used a PHQ-8 sum score of ≥10 to define current depressive symptoms. This well-established cut-off indicates moderate to severe depressive symptoms with clinical relevance, has a sensitivity and specificity for major depression of 88%, and is recommended for general population studies to screen for current depressive symptoms [52, 54].

### Covariates

To prevent potential confounding by sociodemographic factors, chronic illness, and functional limitations, we included age, sex, residential region, immigrant status, long-term chronic conditions, and global activity limitations as covariates in our analysis. To take account of residential region, we differentiated between participants living in the western (old federal states) and those living in the eastern part (new federal states, including Berlin) of Germany. Immigrant status was operationalized with a binary variable distinguishing between participants who were born within the current borders of Germany and those who were not. The presence of long-term chronic conditions and global activity limitations were assessed using the Minimum European Health Module (MEHM) [55].

### Statistical analysis

Pearson’s correlation coefficients were calculated to determine bivariate correlations between the indicators of objective SES and SSS. Principal factor analysis with orthogonal varimax rotation was performed to examine whether SSS loaded on a common latent factor with the other SES variables, and/or whether it had external loadings on a latent depression factor of the PHQ-8 items. The prevalence of current depressive symptoms was estimated across groups of low, medium, and high SES and SSS. Pearson’s chi-square test was used to examine statistically significant differences.

We employed logistic regression models to examine associations of objective SES measures and SSS with current depressive symptoms. To compare the resulting odds ratios (ORs) directly, z-standardized scores of the metric variables of SES and SSS were included in the models. We reversed the z-scores so that ORs greater than 1 indicated increasing odds of depressive symptoms with a lower position in the social hierarchy. The standard regression approach to mediation analysis popularized by Baron and Kenny [56] was used to examine SSS as a mediator between objective SES and depressive symptoms. Accordingly, we estimated a series of regression models: (1) regressing SSS on objective SES, (2) regressing depressive symptoms on objective SES, and (3) regressing depressive symptoms on both objective SES and SSS. All models were adjusted for covariates and standardized coefficients were computed. As the data were cross-sectional, i.e. the social status variables and depressive symptoms were measured during the same survey, the regression and mediation analyses were exploratory in nature and it was not possible to determine the direction of causality between the variables included.

We used weighting factors to account for unequal sampling probabilities and to adjust the distribution of the sample by sex, age, education, and region to match the population in Germany in on 31 December 2012. Analyses were conducted in STATA 14.1 (StataCorp LP, College Station, TX) using the survey data commands to account for the complex sample design. Results were considered statistically significant when *p* < 0.05. All analyses were conducted separately for men and women to identify sex-specific associations and to prevent potential gender bias.

## Results

Table 2 shows the results of the correlation analysis for all objective and subjective socioeconomic status variables. There were strong correlations between the composite index of objective SES and its individual dimensions of education, occupation, and income. The individual SES dimensions were moderately correlated with each other. SSS showed the strongest correlation with the composite index of objective SES (*r* = 0.50), although this correlation was moderate. Among the individual SES dimensions, income showed the strongest correlation with SSS. These patterns were similar for both sexes, although most coefficients were slightly lower for women.

**Table 2:** Correlations between indicators of objective and subjective socioeconomic status, for the total sample and by sex (correlation coefficients).

	SES	Education	Occupation	Income
**Total**				
Education	0.75***			
Occupation	0.76***	0.40***		
Income	0.79***	0.34***	0.41***	
SSS	0.50***	0.34***	0.36***	0.45***
**Men**				
Education	0.78***			
Occupation	0.78***	0.45***		
Income	0.78***	0.37***	0.42***	
SSS	0.53***	0.38***	0.39***	0.47***
**Women**				
Education	0.73***			
Occupation	0.75***	0.38***		
Income	0.79***	0.30***	0.42***	
SSS	0.47***	0.30***	0.32**	0.43***

**p < 0.01;

***p < 0.001;

SES, composite index of objective socioeconomic status; SSS, subjective social status.

Table 3 shows the matrix of rotated factor loadings generated from the factor analysis. The results show a clear two-factor structure for the total sample as well as for men and women. Each of the PHQ-8 depression items had strong loadings on a common latent factor (Factor 1) and the socioeconomic variables loaded strongly on a different factor (Factor 2). SSS loaded on a common latent factor with the other socioeconomic variables and did not show any substantial external loading on the latent depression factor (loadings on Factor 1 were <0.2).

**Table 3:** Rotated factor matrix, for the total sample and by sex (factor loadings from principal factor analysis with orthogonal varimax rotation).

	Total		Men		Women	
	Factor 1	Factor 2	Factor 1	Factor 2	Factor 1	Factor 2
Education	−0.048	0.572	−0.054	0.614	−0.034	0.534
Occupation	−0.066	0.612	−0.056	0.631	−0.086	0.599
Income	−0.115	0.605	−0.117	0.625	−0.109	0.592
SSS	−0.190	0.579	−0.195	0.613	−0.186	0.543
PHQ-8 (1)	0.702	−0.096	0.684	−0.113	0.719	−0.080
PHQ-8 (2)	0.770	−0.105	0.765	−0.117	0.771	−0.096
PHQ-8 (3)	0.563	−0.087	0.585	−0.057	0.540	−0.115
PHQ-8 (4)	0.730	−0.086	0.716	−0.085	0.734	−0.090
PHQ-8 (5)	0.629	−0.093	0.576	−0.074	0.651	−0.114
PHQ-8 (6)	0.701	−0.081	0.667	−0.110	0.720	−0.060
PHQ-8 (7)	0.646	−0.052	0.606	−0.061	0.669	−0.046
PHQ-8 (8)	0.467	−0.098	0.460	−0.120	0.472	−0.081

PHQ-8, Patient Health Questionnaire (8-item depression scale); SSS, subjective social status.

The overall prevalence of current depressive symptoms was 11.0% (95% confidence intervals [CI]: 10.0–12.1%). In women, the prevalence was significantly higher (12.6%; 95% CI: 11.2–14.2%) than in men (9.3%; 95% CI: 8.0–10.8%). Table 4 shows the prevalence of depressive symptoms according to age group and the classified variables of objective SES and SSS. The oldest age group showed the lowest prevalence. The sex difference in the prevalence of depressive symptoms was largest for young adults. For the total sample, there was a statistically significant social gradient in current depressive symptoms for all indicators of objective SES, each favouring the higher socioeconomic groups. The same clear pattern was observed for SSS. The sex-specific analyses showed that this social patterning of depressive symptoms was found in both men and women.

**Table 4:** Prevalence of depressive symptoms according to age group and indicators of objective and subjective socioeconomic status, for the total sample and by sex.

	Total			Men			Women		
	%	(95% CI)	*p*-value	%	(95% CI)	*p*-value	%	(95% CI)	*p*-value
**Age group**									
18–29 years	13.9	(11.8–16.3)		9.5	(6.8–13.2)		18.3	(15.1–21.9)	
30–44 years	12.7	(10.4–15.3)		11.9	(8.9–15.7)		13.4	(10.2–17.5)	
45–64 years	12.2	(10.2–14.6)		10.6	(8.0–13.8)		13.9	(11.2–17.2)	
65+ years	5.5	(3.9–7.7)	0.000	4.2	(2.7–6.6)	0.003	6.5	(4.2–9.9)	0.000
**SES**									
Low	20.5	(17.1–24.4)		23.0	(17.9–28.9)		17.9	(13.8–23.0)	
Medium	10.2	(9.0–11.5)		6.6	(5.2–8.4)		13.3	(11.4–15.3)	
High	4.6	(3.5–6.0)	0.000	4.0	(2.5–6.3)	0.000	5.4	(3.7–7.8)	0.000
**Education**									
Low	16.1	(13.2–19.5)		19.5	(14.6–25.5)		14.2	(10.8–18.3)	
Medium	10.9	(9.7–12.1)		8.5	(6.9–10.4)		13.2	(11.4–15.4)	
High	5.7	(4.4–7.4)	0.000	4.8	(3.0–7.5)	0.000	7.2	(5.0–10.1)	0.026
**Occupation**									
Low	17.7	(14.6–21.2)		15.7	(12.0–20.2)		21.3	(16.2–27.6)	
Medium	10.3	(9.2–11.6)		7.4	(5.7–9.6)		12.4	(10.8–14.2)	
High	6.0	(4.3–8.3)	0.000	5.7	(3.5–9.0)	0.000	6.3	(4.2–9.3)	0.000
**Income**									
Low	20.3	(17.4–23.5)		18.4	(14.3–23.5)		22.0	(18.3–26.1)	
Medium	9.5	(8.2–11.0)		7.7	(6.1–9.7)		11.1	(9.5–13.1)	
High	4.6	(3.2–6.6)	0.000	3.9	(2.0–7.3)	0.000	5.4	(3.3–8.6)	0.000
**SSS**									
Low	18.6	(16.1–21.4)		17.4	(13.7–21.8)		19.8	(16.7–23.3)	
Medium	8.2	(7.0–9.6)		6.3	(4.7–8.3)		9.9	(8.2–12.0)	
High	3.4	(2.0–5.7)	0.000	0.7	(0.2–2.1)	0.000	7.5	(4.1–13.1)	0.000

CI, confidence interval; SES, composite index of objective socioeconomic status; SSS, subjective social status.

The results presented in Table 5 show that both lower objective SES and lower SSS (as indicated by the reversed z-scores of the metric variables) were each associated with higher odds of current depressive symptoms in men and women, after adjusting for sociodemographic factors, long-term chronic conditions, and global activity limitations (Model 1). When objective SES and SSS were mutually adjusted, both remained significantly associated with depressive symptoms (Model 2). The ORs for the associations of objective SES and SSS with current depressive symptoms did not significantly differ from one another (Wald test: total: *p* = 0.722; men: *p* = 0.489; women: *p* = 0.729).

**Table 5:** Adjusted odds ratios of depressive symptoms according to objective socioeconomic status and subjective social status, for the total sample and by sex.

	Model 1^a^		Model 2^b^	
	OR (95% CI)	*p*-value	OR (95% CI)	*p*-value
**Total**				
SES_rz_	1.66 (1.42−1.95)	0.000	1.39 (1.16−1.65)	0.000
SSS_rz_	1.67 (1.46−1.91)	0.000	1.46 (1.25−1.69)	0.000
**Men**				
SES_rz_	1.78 (1.37−2.32)	0.000	1.38 (1.03−1.85)	0.032
SSS_rz_	1.86 (1.49−2.32)	0.000	1.62 (1.26−2.09)	0.000
**Women**				
SES_rz_	1.61 (1.36−1.89)	0.000	1.41 (1.16−1.72)	0.001
SSS_rz_	1.53 (1.30−1.81)	0.000	1.33 (1.09−1.62)	0.005

OR, odds ratio; CI, confidence interval; SES, composite index of objective socioeconomic status; SSS, subjective social status.

_rz_ reversed z-score (standardized reversed variable).

^a^ Separate regression models, adjusted for age, age^2^, residential region, immigrant status, chronic conditions, global activity limitations (plus adjustment for sex and sex*age in the total sample).

^b^ Mutual adjustment of SES and SSS, adjusted for age, age^2^, residential region, immigrant status, chronic conditions, global activity limitations (plus adjustment for sex and sex*age in the total sample).

**Fig 1:**
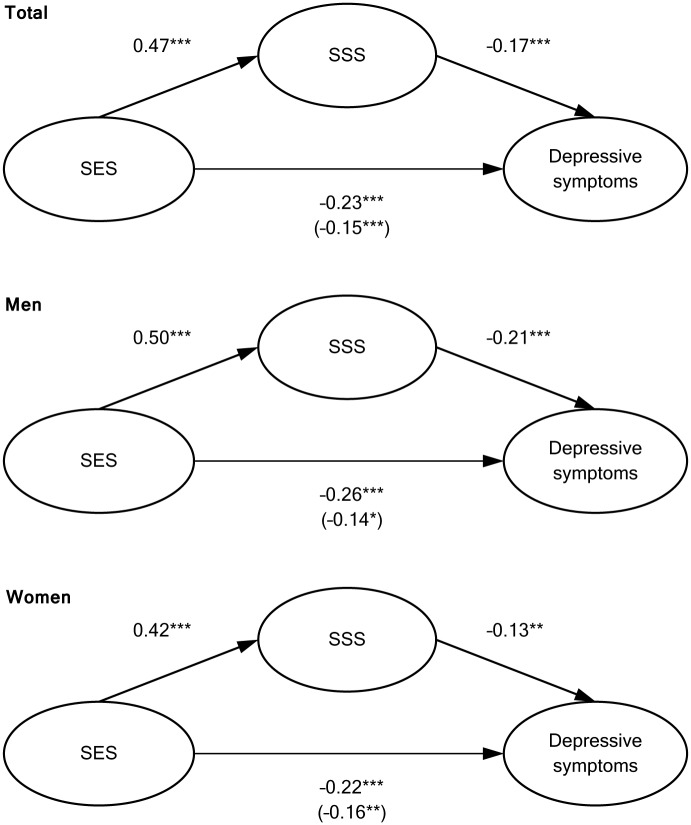
Mediation analysis of the association between objective socioeconomic status (SES) and depressive symptoms as mediated by subjective social status (SSS). Standardized coefficients, adjusted for age, age^2^, residential region, immigrant status, chronic conditions, global activity limitations (plus sex and sex*age in the total sample). Coefficients for direct associations in parentheses. *p < 0.05; **p < 0.01; ***p < 0.001.

Fig 1 shows the results of the mediation analysis, adjusted for all covariates. The series of regression models showed that all criteria for mediation were met: (1) objective SES (independent variable) was associated with SSS (mediator variable), (2) objective SES was associated with depressive symptoms (dependent variable), (3) SSS was associated with depressive symptoms while adjusting for objective SES, and (4) the coefficients for the association between objective SES and depressive symptoms decreased significantly when SSS was additionally adjusted for (Wald test: total: *p* < 0.001; men: *p* = 0.001; women: *p* < 0.001). Correspondingly, there was a significant indirect association of objective SES with depressive symptoms as mediated through SSS (standardized coefficient for indirect association: total: −0.08, *p* < 0.001; men: −0.12, *p* < 0.001; women: 0.06, *p* = 0.006).

Table 6 shows the results of the analyses to examine the relative importance of the individual dimensions of objective SES. Each dimension was significantly associated with current depressive symptoms after adjusting for sociodemographic factors, long-term chronic conditions, and global activity limitations (Model 1). After mutual adjustment of the individual dimensions, lower occupational status and lower income remained associated with higher odds of depressive symptoms, whereas the association between education and depressive symptoms was no longer statistically significant (Model 2). Model 3 additionally included SSS, which showed a significant association with depressive symptoms when education, occupation, and income were held constant. Simultaneously, in the total sample, the relations of occupational status and income with depressive symptoms remained significant after adjusting for SSS. Stratified by sex, income became insignificant in both sexes and occupation became marginally insignificant in men after adding SSS to the model. Overall, the association of income with depressive symptoms attenuated more than the association of occupational status with depressive symptoms when SSS was added to the model.

**Table 6:** Adjusted odds ratios of depressive symptoms according to individual dimensions of objective socioeconomic status and subjective social status, for the total sample and by sex.

	Model 1^a^		Model 2^b^		Model 3^c^	
	OR (95% CI)	*p*-value	OR (95% CI)	*p*-value	OR (95% CI)	*p*-value
**Total**						
Education_rz_	1.28 (1.12−1.46)	0.000	1.04 (0.90−1.20)	0.587	0.98 (0.85−1.13)	0.773
Occupation_rz_	1.50 (1.32−1.72)	0.000	1.34 (1.15−1.56)	0.000	1.28 (1.09–1.50)	0.003
Income_rz_	1.51 (1.31−1.73)	0.000	1.33 (1.16−1.53)	0.000	1.18 (1.01–1.37)	0.034
SSS_rz_					1.45 (1.25–1.69)	0.000
**Men**						
Education_rz_	1.32 (1.04−1.67)	0.023	1.04 (0.79−1.37)	0.771	0.95 (0.72−1.25)	0.714
Occupation_rz_	1.54 (1.25−1.90)	0.000	1.36 (1.06−1.76)	0.018	1.28 (0.99−1.66)	0.064
Income_rz_	1.60 (1.27−2.02)	0.000	1.42 (1.11−1.81)	0.006	1.19 (0.92−1.54)	0.174
SSS_rz_					1.62 (1.26−2.09)	0.000
**Women**						
Education_rz_	1.28 (1.08−1.51)	0.004	1.06 (0.89−1.26)	0.505	1.02 (0.86−1.22)	0.811
Occupation_rz_	1.48 (1.28−1.71)	0.000	1.33 (1.13−1.57)	0.001	1.29 (1.09−1.52)	0.004
Income_rz_	1.45 (1.24−1.71)	0.000	1.27 (1.06−1.52)	0.010	1.16 (0.95−1.41)	0.136
SSS_rz_					1.33 (1.09−1.62)	0.005

OR, odds ratio; CI, confidence interval; SSS, subjective social status.

_rz_ reversed z-score (standardized reversed variable).

^a^ Separate regression models, adjusted for age, age^2^, residential region, immigrant status, chronic conditions, global activity limitations (plus adjustment for sex and sex*age in the total sample).

^b^ Mutual adjustment of the single dimensions of objective SES, adjusted for age, age^2^, residential region, immigrant status, chronic conditions, global activity limitations (plus adjustment for sex and sex*age in the total sample).

^c^ Model 2 plus subjective social status.

## Discussion

Using national data from Germany, this study is among the first to investigate associations of both objective SES indicators and SSS with the presence of depressive symptoms in adults. Our findings demonstrate that lower objective SES and lower SSS are independently associated with current depressive symptoms. Further, the results suggest that the association between objective SES and depressive symptoms is partially mediated through perceptions of social status, as represented by SSS, for both men and women. Education, occupation, and income were each associated with current depressive symptoms, although income and occupation showed stronger associations with depressive symptoms than education. When all status indicators were mutually adjusted, occupational status, income, and SSS remained independently associated with symptoms of depression.

### Strengths and limitations

To the best of our knowledge, the associations of objective SES and SSS with depressive symptoms have never before been studied using national data from Germany. Owing to the sample design and the weighting factors used to adjust for survey non-response, it is possible to draw conclusions for the adult population in Germany from our results. As data were collected using self-administered questionnaires, social desirability bias should have been low, because this type of bias mainly occurs when interviewers are involved in the data collection process [57, 58]. Recall bias should also have been low, as the measures were based on questions referring to the present or the last 2 weeks. The metric scales of the SES and SSS measures and the use of their standardized values in the regression models enabled adequate comparison of their associations with depressive symptoms.

Several limitations of our study should be taken into account when interpreting the results. Because the data were cross-sectional, we could not infer any causality or establish the causal direction of the associations. We assumed that adults’ socioeconomic situation affects their risk of depressive symptoms, but the association is likely to be bidirectional. For example, data from statutory health insurances and pension funds in Germany show that mental disorders, especially depression, are one of the main reasons for work inability and early retirement [59]. This may partly explain the cross-sectional association we found between SES and depressive symptoms. With regard to SSS, the prospective Whitehall II study has effectively shown that low SSS predicts a future decline in mental health [36]. Longitudinal data from Taiwan demonstrate that higher SSS is associated with a lower risk of depressive symptoms, even after controlling for baseline depressive symptoms and other baseline characteristics [60]. Schubert et al. [61] report experimental evidence suggesting that SSS has causal effects on depressive thinking among students. These studies support the causal direction assumed in our study. However, other longitudinal data suggest that associations between SSS and health may arise from effects operating in both directions [62]. An effect of depressive symptoms on SSS is plausible because people with depressed mood supposedly have a worse appraisal of their socioeconomic situation than do people without depressed mood.

The limitations of the cross-sectional design apply also to the mediation analysis. The statistically significant mediation result is not irrefutable evidence of causal mediation; it only suggests that the hypothesized mediation model is compatible with the data used. Requirements regarding model specification for mediation analysis—for example, no omitted variables and no reverse causality (i.e. correct temporal order)—were not met by the cross-section data we used. This can lead to biased estimates and improper inferences and must be kept in mind when interpreting the results. To overcome these limitations in the future, prospective data are needed to establish the direction of effects, and to address questions of causality and causal mediation.

Further, it should be noted that the PHQ-8 is a screening instrument that assesses current depressive symptoms but does not provide a differential diagnosis of depression. Many people who are positively screened on this measure may have subthreshold depressive symptoms [7]. Other limitations arise from the relatively low response rate. Systematic non-response may have led to selection bias, which could have affected our results. For example, the fact that women in the sample had a slightly higher occupational status on average than men may be an indication of sample selectivity; women of lower occupational status could have had a lower response to the survey and therefore be under-represented in the sample. However, as the literature suggests that there is no clear relation between response rate and representativeness of response [63], a low response does not necessarily lead to strong selection or weak external validity in general. Nonetheless, to minimize potential selection bias, we used weighting factors to adjust the net sample to match the population in Germany. Residual selectivity may, however, have remained.

### Previous findings and potential pathways

Our results support many previous findings indicating that low objective SES is associated with depressive symptoms and depressive disorders [5–7, 64]. The findings also accord with those of earlier studies suggesting that income is more strongly associated with depressive symptoms than is education. However, findings related to the relative importance of occupation are less consistent [65, 66]. This could be because of substantial between-studies differences in classifying and ranking occupations. Our findings clearly indicate that low occupational status is an independent socioeconomic determinant of depressive symptoms in adults. Low control at work and the imbalance between effort spent and reward received in working life have been identified as essential mediators of this relationship in previous research [18, 67]. With regard to our findings, however, it is worth noting that the occupational status measure used in our study was operationalized as a household characteristic. It is accordingly rather a measure of the occupational social prestige of the participants' household than a measure of the participants' individual occupational position. Hence, social disadvantages or negative emotions that arise from low social prestige may also be a mediating factor in the association we found between lower occupational status and depressive symptoms.

Our finding that the association of income with depressive symptoms attenuates and almost disappears when SSS is controlled for indicates that the pathway linking income to depressive symptoms may be a psychosocial rather than a material one. SSS may reflect perceptions of financial stress, thereby explaining in part the association between income and depressive symptoms. This is supported by previous research suggesting that perceived financial stress (i.e. worries about having enough money for daily needs) acts as a psychosocial stressor linking low income with symptoms of depression [68].

In accord with recent research from several countries [24, 32, 35, 69], our findings demonstrate that lower SSS is associated with poorer mental health over and above the associations with objective SES measures. Such an independent association of SSS was previously found for depressive symptoms among adults in the U.S., England, and Hong Kong [70–74]. Our study is the first to examine this association representatively for Germany, and the findings demonstrate that the association also exists in a Central European country in times of good economic development over the preceding years, low unemployment rates, and a relatively high level of average SSS compared to other European countries. To the best of our knowledge, the study by Demakakos et al. [72] is the only study to also explicitly explore whether SSS mediates the associations of objective SES measures with depressive symptoms in adults. In line with our findings, they found that SSS partially mediates these associations; however, as they used a sample of older adults and other indicators of objective SES, our results cannot directly be compared with theirs. The study by Collins & Goldman [60] suggests that half or more of the cross-sectional association between SSS and mental health could be a result of endogeneity; i.e., SSS may capture not only perceptions of one’s social standing but also aspects of (mental) health, which could partly explain the strong cross-sectional association. Accordingly, the cross-sectional association we found between SSS and depressive symptoms may also be overestimated.

A low SSS indicates that people perceive themselves as socioeconomically disadvantaged in comparison with others; this suggests that low SSS is a result of social comparison processes and represents an operationalization of perceived relative deprivation. Perceptions of relative deprivation may lead to frustration, shame, feelings of inferiority, and stress, which in turn can have adverse implications for health [75–77]. Consequently, perceptions of relative deprivation may be part of a psychosocial pathway linking inequalities in socioeconomic resources to inequalities in health.

Research has often identified stress-related neuroendocrine processes as mechanisms underlying the relationship between perceptions of relative deprivation and poor health [78, 79]. Goodman et al. [80] applied this idea to a model of the relationships between objective SES, SSS, and obesity by drawing on knowledge about physiological stress responses. They suggest that SSS mediates the relationship between low SES and obesity through the activation of the hypothalamic–pituitary–adrenal (HPA) axis, a part of the neuroendocrine system controlling responses to stress. The activation of the HPA axis, in turn, can result in weight gain and lead to obesity. This idea is supported by empirical evidence showing that low SSS is associated with higher physiological stress markers [79, 81, 82]. Similarly, a neuroendocrine pathway may link SSS to depressive symptoms, as evidence suggests that the stress-related dysregulation of the HPA axis also predicts the onset and recurrence of depression [83].

An alternative explanation of why low SSS predicts ill-health independently from objective SES is that SSS may be a more comprehensive measure of people’s socioeconomic situation than traditional objective SES measures. Singh-Manoux and colleagues [36] speculate that SSS might not only represent a ‘cognitive average’ of current socioeconomic circumstances, but might also take account of past trajectories and perceived future prospects. Moreover, when people rank themselves on the SSS ladder, they might refer to socioeconomic factors other than (or additional to) formal education, occupation, and income, such as financial debts, assets, inheritances, home ownership, or advanced vocational training [69].

## Conclusions

Since the concept of SSS has been introduced to health research, a rapidly growing body of studies has investigated its relationships with a broad range of health indicators in different populations [23, 24]. However, evidence from European countries is still scarce. Using data from adults in Germany, this study expands the research on this topic and shows that lower self-perceived position in the hierarchy of a modern society is associated with the presence of depressive symptoms, independently of the social position assigned to people based on their education, occupation, and income. The results therefore indicate that perceptions of social disadvantage may be involved in the pathogenesis of adult depression. The findings further suggest that social comparison processes may be part of a psychosocial pathway linking people’s socioeconomic situation with depressive symptoms. Our findings, and those from previous studies, suggest that SSS is an indicator capturing aspects of social inequality that have implications for health and that are not captured by traditional measures of SES. Thus, SSS is a convenient and valid measure that can meaningfully complement conventional SES measures, and which has the potential to provide relevant insights into the nature of health inequalities and their underlying mechanisms. In the future, longitudinal data will be required to establish the direction of effects and to address questions of causality.
